# “Exploring Surgical Techniques for Rhinophyma: A Detailed Analysis of Cases”

**DOI:** 10.1155/crot/4284687

**Published:** 2026-01-20

**Authors:** Theodora Ligomenou, Eirini Nikolaidou, Argyro Pipinia, Zafiris Fachouris, Loukas Stefanou, Glykeria Pantazi

**Affiliations:** ^1^ Department of Plastic, Reconstructive and Hand Surgery & Burn ICU, G. Papanikolaou General Hospital, Thessaloniki, Greece, gpapanikolaou.gr

**Keywords:** case report, dermal substitutes, flap reconstruction, rhinophyma, surgery

## Abstract

“Rhinophyma” comes from the Greek words “rhis,” meaning nose, and “phyma,” meaning growth, which reflects its clinical presentation; overgrowth of sebaceous glands results in disfigurement or even nasal obstruction, in more severe cases. Nonsurgical treatments are the standard care, with dermabrasion, laser therapy, and ablative treatments being the most commonly preferred options. For advanced or recurrent cases, surgical intervention is the gold standard. Various surgical techniques have been described, including skin grafts, flaps, and skin substitutes. Surprisingly, there are only a few case reports in the literature regarding the surgical management of rhinophyma. We present two cases of severe and recurrent rhinophyma treated surgically in one stage procedure under local anesthesia by two different surgical techniques, one flap reconstruction and one dermal substitute and split‐thickness skin graft reconstruction. Both patients had breathing difficulties, which were resolved after the surgical intervention. No major complications were detected during the 2 years of follow‐up. Patients were satisfied with the aesthetic and functional outcomes. The surgical approach of rhinophyma should be the standard of care for recurrent cases or cases with nasal obstruction. Different surgical techniques have been described. As long as surgical planning is concerned, it is essential to consider practical and clinical factors such as patient’s preferences, one or more stages of reconstruction, healing time, safety, and recurrence rates.

## 1. Introduction

Rhinophyma is a benign skin condition affecting the nose, characterized by hyperplasia of the sebaceous glands [1]. The term “rhinophyma” comes from the Greek words “rhis,” meaning nose, and “phyma,” meaning growth, which reflects its clinical presentation. This condition primarily affects male Caucasians aged 40–80 and is often associated with phymatous rosacea, a common and persistent skin condition of the face [2]. In its early stages, rosacea typically presents with mild swelling, redness (erythema), and broken blood vessels (telangiectasia). However, in its severe forms, it can lead to significant deformation of the nasal tip, which may even cause airway obstruction [1, 2]. While malignant transformation is rarely reported, the potential link between rhinophyma and carcinoma remains uncertain [3].

Currently, nonsurgical treatments are the standard care for rhinophyma, with dermabrasion, laser therapy, and ablative treatments being the most commonly preferred options [1]. For advanced or recurrent cases, surgical intervention is the gold standard. Various surgical techniques have been described, including skin grafts, flaps, and skin substitutes. Surprisingly, there are only a few case reports in the literature regarding the surgical management of rhinophyma [4, 5]. The aim of our case series is to present two cases of giant rhinophyma that were successfully treated using different surgical techniques.

## 2. Case Presentation

### 2.1. Case 1

**Figure 1:**
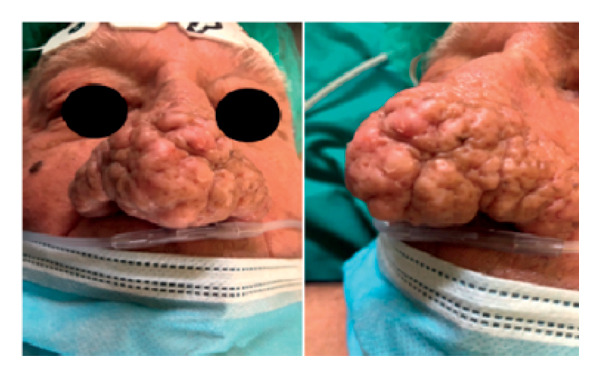
Preoperative face and profile photo of a 76‐year‐old male patient with breathing difficulties caused by a giant rhinophyma.

**Figure 2:**
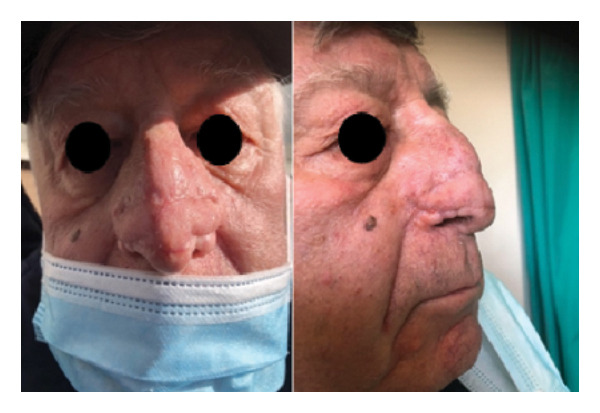
Postoperative face and profile photo of a 76‐year‐old male patient after excision of a giant rhinophyma and reconstruction using a bilateral nasolabial flap. Both the function and the form have been restored.

A 76‐year‐old male patient presented with breathing difficulties caused by the gradual enlargement of his nose over the years (Figure 1). He had previously undergone fractionated CO_2_ laser resurfacing therapies, but the results were unsatisfactory. After experiencing multiple recurrences, surgical intervention was deemed necessary. Under local anesthesia, the surgical team completely excised the rhinophyma down to the underlying cartilage using a surgical knife. The resulting nasal defect was reconstructed using bilateral nasolabial flaps. During a follow‐up appointment 3 months later, the postoperative cosmetic outcome was deemed satisfactory (Figure 2).

### 2.2. Case 2

**Figure 3:**
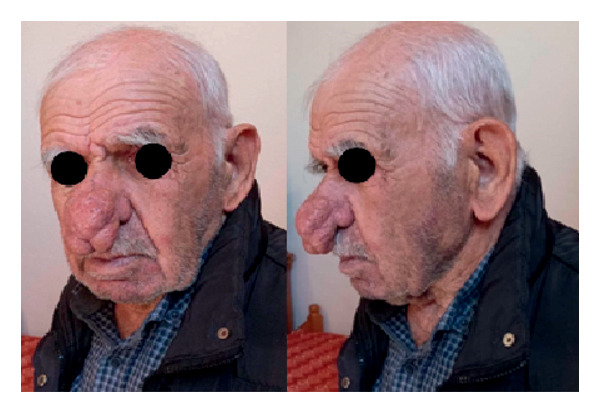
Preoperative face and profile photo of an 82‐year‐old male patient with a giant rhinophyma causing nasal obstruction.

**Figure 4:**
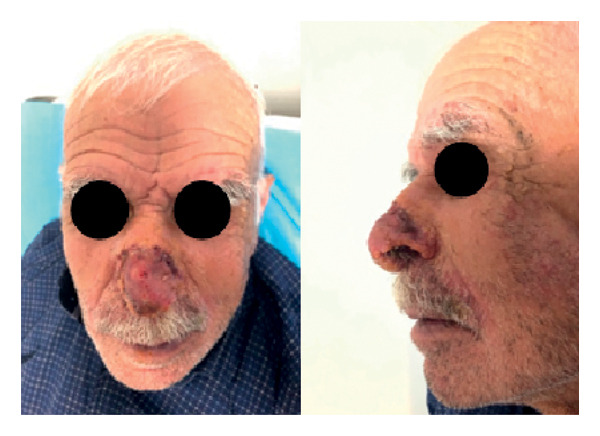
Postoperative face and profile photo of an 82‐year‐old male patient after excision of a giant rhinophyma and reconstruction using MatriDerm and split‐thickness skin grafts. The aesthetic and functional results were satisfactory at the 2‐month follow‐up.

An 82‐year‐old male patient presented with a giant rhinophyma that was causing nasal obstruction (Figure 3). Due to the size of the rhinophyma, a surgical approach was deemed necessary. Under local anesthesia, the rhinophyma was surgically removed and sent for histopathological analysis. The resulting defect was covered with MatriDerm, a bovine collagen–elastin template that serves as a dermal substitute scaffold. In addition, split‐thickness skin grafts were taken from the postauricular area to cover the defect (Figure 4).

## 3. Discussion

We present two cases of rhinophyma that were treated surgically. In the first case, the patient experienced multiple recurrences after undergoing CO_2_ laser resurfacing and other ablative techniques. Despite initially promising results, the condition returned a few months later in a more aggressive form, leading to nasal obstruction and breathing difficulties. As the patient sought a more permanent solution, a surgical approach was recommended. Our surgical goal was to completely excise the rhinophyma and to reconstruct both function and appearance. We identified the bilateral nasolabial flap as a suitable option due to its excellent blood supply, ease of harvesting, and potential for improved cosmetic and functional outcomes. In the second case, we opted for an alternative surgical approach that involved thorough excision followed by the application of a collagen–elastin matrix for dermal replacement. This was combined with the use of an autologous split‐thickness skin graft. In the first case, the patient agreed to reconstruct the defect using local flaps. In contrast, the second patient was uncomfortable with the idea of having a flap donor site on the face. Instead, we proposed the use of a dermal substitute to create a neodermis, which helped improve the quality of the final skin contour and reduced the risk of secondary skin contraction. This method eliminates the need for local flaps. At the 2‐year follow‐up, the patients were satisfied with breathing and the aesthetic result, and no major complications were detected.

Both of our techniques were based on the following considerations: Thorough and deep excision of the cartilage was essential for the complete removal of the affected tissue, thus minimizing the likelihood of recurrence. In addition, since the cartilage framework was not distorted by the rhinophyma in either case, there was no need for cartilage reconstruction. Both reconstructive techniques eliminate the necessity of obtaining donor sites for full‐thickness skin grafts. Furthermore, they can be applied under local anesthesia, in one single stage.

## 4. Conclusion

Rhinophyma is an uncommon condition with an unclear underlying mechanism. It is crucial to address this disfiguring disorder since patients often experience psychological distress and respiratory problems, particularly when thickening of the alar region obstructs the external nasal valves. The surgical approach of rhinophyma should be the standard of care for recurrent cases or cases with nasal obstruction. Different surgical techniques have been described [3–5]. When proceeding to surgical planning, it is essential to consider practical and clinical factors such as patient’s preferences, one or more stages of reconstruction, healing time, safety, and recurrence rates.

## Consent

An informed consent was obtained preoperatively and for photographic material release.

## Conflicts of Interest

The authors declare no conflicts of interest.

## Funding

This research received no specific grant from any funding agency in the public, commercial, or not‐for‐profit sectors.

## Data Availability

The data that support the findings of this study are available from the corresponding author upon reasonable request.
